# Comparison Study between Conventional Sequence and Slice-Encoding Metal Artifact Correction (SEMAC) in the Diagnosis of Postoperative Complications in Patients Receiving Lumbar Inter-Body Fusion and Pedicle Screw Fixation Surgery

**DOI:** 10.1371/journal.pone.0163745

**Published:** 2016-10-06

**Authors:** Sol Bee Han, Young Cheol Yoon, Jong Won Kwon

**Affiliations:** Department of Radiology, Samsung Medical Center, Sungkyunkwan University School of Medicine, Seoul, Korea; Northwestern University Feinberg School of Medicine, UNITED STATES

## Abstract

**Background and Purpose:**

Slice-Encoding Metal Artifact Correction (SEMAC) sequence is one of the metal artifact reduction techniques of anatomical structure, but there has been no report about evaluation of post-operative complications. The purpose of this article is to compare the anatomical visibility between fast spin echo (FSE) and FSE-SEMAC and to evaluate the additional value of FSE-SEMAC in diagnostic confidence of the complications.

**Materials and Methods:**

We conducted a retrospective study with 54 patients who received lumbar spinal surgery and MR images including FSE-SEMAC. For the semi-quantitative evaluation, the visibility of anatomical structures (neural foramen, bone-inter-body cage interface, central canal, nerve root in epidural space, back muscle, and bone-pedicle screw interface) was evaluated. For qualitative evaluation, we evaluated FSE and FSE with FSE-SEMAC independently, and recorded the diagnostic confidence level of post-operative complications. Generalized estimating equation regression analysis was used for statistical analysis, and a weighted kappa was used for inter-observer agreement.

**Results:**

Scores of 6 imaging findings with FSE-SEMAC were significantly higher than that of FSE (*P*-value < .0001). Inter-observer agreements show good reliability (weighted kappa = 0.45–0.75). Both reviewers deemed 37 (reviewer 1) or 19 more (reviewer 2) post-operative complications with FSE plus FSE-SEMAC, compared to FSE only. Except for central canal stenosis (*P*-value = .2408), diagnostic confidence level for other post-operative complications were significantly higher with FSE plus FSE-SEMAC (*P*-value = .0000) than FSE.

**Conclusions:**

FSE-SEMAC significantly reduces image distortion, compared to FSE sequence in 3.0-T MR. Also, diagnostic confidence for post-operative complications was higher when FSE with additional FSE-SEMAC compared to FSE only.

## Introduction

Metallic spinal implants are frequently used in spinal surgery for decompression and spinal fusion due to conditions such as herniated intervertebral disc (HIVD), spinal stenosis, tumor, infection, trauma, and congenital anomaly [1]. Local complications after surgical procedures that use spinal instrumentation include loosening, prosthetic or peri-prosthetic fracture, migration, infection, heterotopic bone formation and osteolysis. Radiological evaluation can help to determine factors related to morbidity and mortality [2]. MRI is usually used to evaluate postoperative complications in patients with sustained back pain, in the case of suspicious adverse events related to surgery, and as a superior method for assessing the anatomical structures compared with simple radiography or computed tomography (CT); however, metal artifacts can limit the evaluation of disease with MRI [1,3].

The slice-encoding metal artifact correction (SEMAC) technique enhances evaluation for metal artifacts via robust encoding of each excited slice against metal-induced field inhomogeneity which is achieved by extending a view-angle-tilting (VAT) spin-echo sequence with additional z-phase encoding [4]. Several recent studies have revealed that SEMAC can reduce metal artifacts of spine, knee and hip MR imaging in patients with metal implants [2,5–9]. Existing clinical studies using SEMAC studied 1.5 T- MR Imaging in patients with total hip arthroplasty (THA) and/or total knee arthroplasty (TKA) [7,9]. There are reports which discuss the usefulness of SEMAC on spinal MR imaging [8,10]. Among them, Lee et al. have quantitatively analyzed the usefulness of fat-saturated T2 weighted sequence with SEMAC at 3T- MR imaging in minimizing metal prosthesis related MR artifacts in patients with spinal prostheses [10]. However, many patients with spinal prosthesis still complain residual or recurrent pain that may be associated with postoperative complications. In our knowledge, there has been no report, which evaluates postsurgical complication with semi-qualitative analysis about diagnostic confidence. Furthermore, for the usefulness of SEMAC with gadolinium (Gd)-enhanced T1-weighted images for the diagnosis of post-surgical complications on spine MR imaging, existing clinical studies only compared T2- weighted images with SEMAC and prioritized anatomical visualization in SEMAC [11–14].

Therefore, we conducted a semi-quantitative evaluation for the visibility of anatomical structures and qualitative evaluation for the diagnostic confidence of post-operative complications with FSE-SEMAC images in 3.0-T MR. The purpose of this study was to compare the results of both semi-quantitative and qualitative post-operative evaluation with metallic hardware using conventional fast spin echo with and without SEMAC with a 3.0-T MR scanner.

## Materials and Methods

### Ethics Statement

Our institutional review board (Samsung Medical Center) approved this retrospective study (No.2014-04-064) and the requirement to obtain written informed consent to participate in this study was waived.

#### Patients

**Fig 1:**
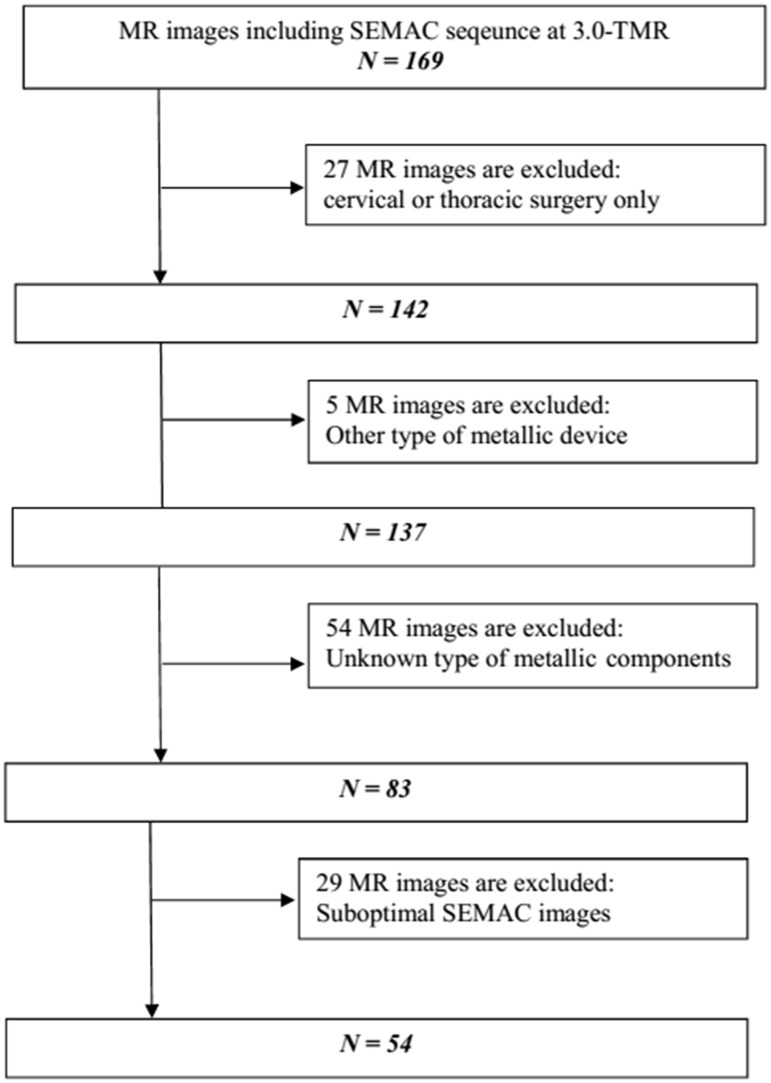
Study design and inclusion and exclusion criteria. Initially, we included 169 MR imaging sets obtained from patients who underwent spinal surgery using instrumentation.

We searched electronic medical records and included 169 consecutive patients who underwent pedicle screw insertion with or without inter-body fusion, as well as postoperative MR imaging including FSE-SEMAC sequence, between March 2011 and December 2012. We eliminated patients who had only cervical or thoracic spine surgery, metallic devices made of substances other than titanium alloy, or any type of unknown metallic device. We also excluded patients with only one plane of FSE-SEMAC sequence imaging or an axial plane of post-contrast T1 FSE-SEMAC sequence with insufficient coverage of the targeted spinal level. We finally enrolled 54 patients with 54 MR imaging sets suitable for evaluation; all patients underwent thoracolumbar or lumbosacral spinal surgery using titanium alloy (age range, 23–85 years, mean age of 62.0 ± 12.93 years, 18 men and 36 women; Fig 1). 52 patients underwent both inter-body fusion and pedicle screw fixation. Two patients had only pedicle screw fixation without inter-body fusion. Degenerative spinal diseases including central or neural foraminal stenosis and spondylolisthesis were common causes of spinal surgery (48 of 54 patients, 89%). Other causes include metastasis, osteosarcoma, malignant peripheral nerve sheath tumor, giant cell tumor, infectious spondylitis, and unstable burst fracture in remained 6 patients, respectively. The mean interval between surgery and MRI examination was 55.5 months (1.1–182.4 months).

### MRI Acquisition

All MR images were obtained with a clinical whole-body 3.0-T MRI system (Achieva 3.0 T TX; Philips Healthcare, Best, Netherlands). Fast spin echo (FSE) T1-weighted, T2-weighted, and post-contrast T1-weighted images were obtained in the axial and sagittal planes. Pre-contrast sagittal T2-weighted FSE-SEMAC images and axial post-contrast T1-weighted FSE-SEMAC images were also obtained. Parameters of each sequence are explained in Table 1. A SEMAC factor of 11 was used in both axial and sagittal FSE-SEMAC images. SEMAC factor is defined as s the number of additional Z phase encoding steps. Slice-corrected MR images were reconstructed from raw images after the SEMAC scan in the MR console. Axial post-contrast T1-weighted FSE-SEMAC images were obtained for two consecutive intervertebral disc levels, including the level of inter-body cage insertion or pedicle screw insertion. If there were more than two levels of inter-body fixation or pedicle screw insertion, one of our musculoskeletal radiologists reviewed the plain radiograph and clinical symptoms and chose the appropriate MRI acquisition levels.

**Table 1:** MRI parameters.

	Pre-contrast T1 weighted images		Pre-contrast T2 weighted images			Post-contrast T1 weighted images		
	axial	sagittal	axial	sagittal	sagittal with FSE-SEMAC	axial	sagittal	axial with FSE-SEMAC
TR (ms)	540	400	4740	3160	1766	540	400	700
TE (ms)	10	10	120	120	120	10	10	12
ETL	6	6	30	29	29	6	6	7
FOV (cm)	22	28	22	28	25	22	28	25
Thickness (mm)	4	4	4	4	4	4	4	4
Matrix (acquisition)	340 x 335	552 x 252	340 x 329	652 x 250	276 x 271	340 x 335	552 x 252	276 x 271
NSA	3	4	2	1	1	3	4	1
Bandwidth	804	596	817	534	793	804	596	798
Acquisition time(s)	200	211	161	164	310	200	211	390

TR = repetition time; TE = echo time; ETL = echo train length; BW = receiver band width; FOV = field of view; NAS = number of signal average.

### Imaging Evaluation

Two musculoskeletal radiologists (Y.Y.C. and K.J.W., with 10 and 9 years of experience evaluating spine MRI, respectively) evaluated images independently. To minimize inter-observer variation, among enrolled 54 patients, 10 MR images that were selected with randomized manner were evaluated for training of the semi-quantitative and qualitative evaluation before using new grading system.

### Semiquantitative Analysis

We evaluated the visibility of six anatomical structures (neural foramen, central canal, nerve root in epidural space, back muscle, bone-inter-body cage interface, and bone-pedicle screw interface) for semi-quantitative evaluation using new grading system, rather than quantitative method using comparison of numeric value. One author (H.S.B) displayed a conventional imaging set (T2 weighted sagittal and Gd-T1-weighted axial) or a SEMAC set (FSE-SEMAC-T2-weighted sagittal and FSE-SEMAC-Gd-T1-weighted axial) on a picture archiving and communication system (RA1000; General Electric Medical Systems, Milwaukee, WI, USA) in a random order. Then, two reviewers scored the visibility of the six anatomical structures at the same time but individually. Any discussion or comment was not allowed between the two reviewers. And only the author who didn’t score (H.S.B) could scroll MR images. Sagittal images obtained at the level of the neural foramen under the pedicle screw and both the upper and lower bone-inter-body cage interfaces were used. The axial image obtained at the level of the neural foramen was used to evaluate the nerve root in the epidural space, and the axial images obtained at the level of the pedicle screw were used to evaluate the visibility of the central canal, back muscles, and bone-pedicle screw interface.

**Fig 2:**
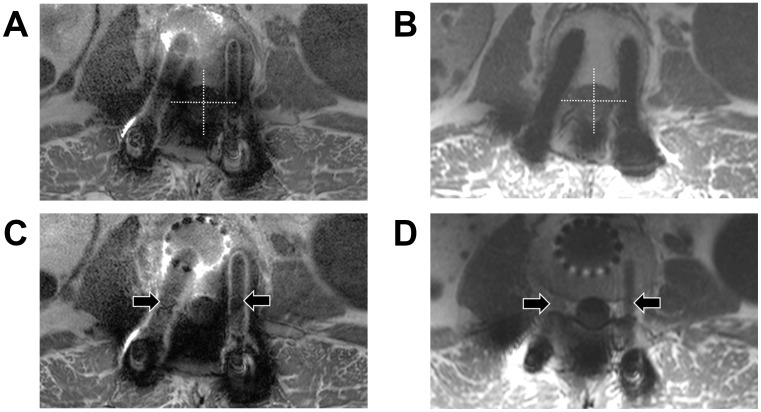
Visibility score of central canal and nerve roots in epidural space in axial images. These four axial images are obtained from a 68 year old male who underwent inter-body fusion at L4-5 and posterior instrumentation at L2-L5 level due to loosening of pedicle screws and infection of previous metallic implants which were inserted for L4 compression fracture. The time interval between surgery and the spine MRI was 5 years and 10 months. Axial fast spin echo T1-weighted image after administration of contrast media (a) shows grade 0,1,0,0 (observer 1) and 0,0,0,0 (observer 2) of visibility of each quadrant of central canal at L3 level. (Right upper, left upper, right lower and left lower, respectively) FSE-SEMAC T1- weighted axial image after administration (b) of contrast media demonstrated grade 2,2,1,2 (observer 1) and grade 2,1,2,1 (observer 2) of each quadrant of central canal. Axial fast spin echo T1-weighted image after administration of contrast media (c) shows grade 0,0 (observer 1) and grade 1,1 (observer 2) in right and left nerve root (arrows), respectively. After application of SEAMC technique, Axial FSE-SEMAC T1- weighted image after administration of contrast media (d) demonstrated grade 3,2 (observer 1) and grade 2,2 (observer 2) in right and left nerve root (arrows), respectively.

**Fig 3:**
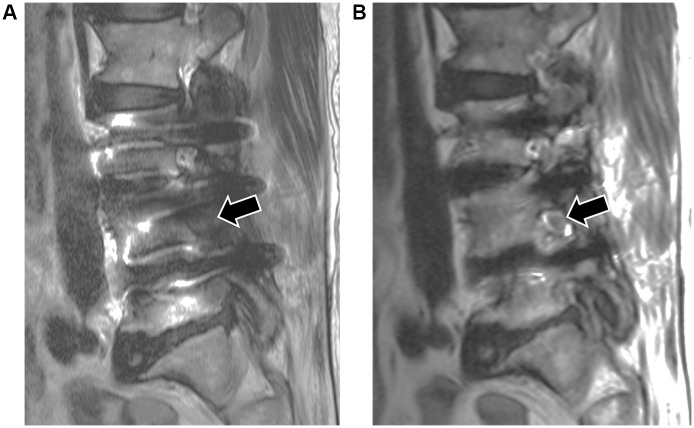
Visibility score of neural foramen and diagnostic confidence of neural foraminal stenosis in sagittal images. These two sagittal images are obtained from a 75 year old female who underwent inter-body fusion at L3-4 and L4-5 and posterior instrumentation at L3-L5 level due to spinal stenosis. The time interval between surgery and the spine MRI was 2 years and 6 months. (a) Sagittal Fast spin echo T2 -weighted image shows grade 0 (observer 1) and 0 (observer 2) of visibility of neural foramen at the left L4-5 (arrow). (b) Sagittal FSE-SEMAC T2- weighted image shows grade 3 (observer 1) and 2 (observer 2) of visibility of neural foramen at the same level (arrow). After application of FSE-SEMAC sequence, diagnostic confidence level of neural foraminal stenosis was changed from grade 3 (inconclusive) to grade 1 (definitely absent) in both reviewers.

**Fig 4:**
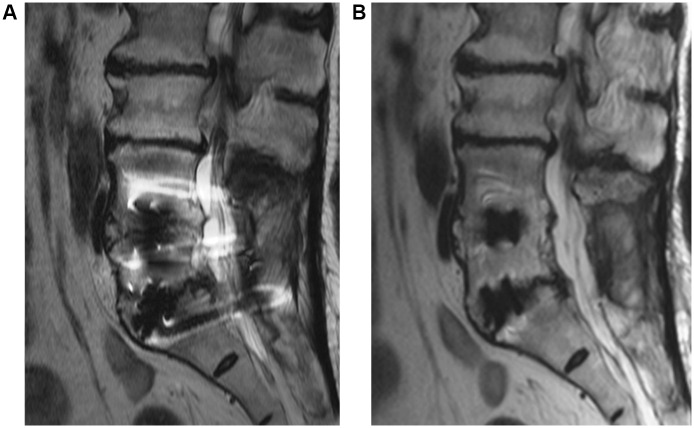
Visibility score of bone-cage interface and diagnostic confidence of inter-body non-union in sagittal images. These two sagittal images are obtained from a 72 year old female who underwent inter-body fusion at L4-5 and L5-S1 and posterior instrumentation at L4-S1 level due to spinal stenosis. The time interval between surgery and the spine MRI was 1 years and 3 months. (a) Sagittal Fast spin echo T2 weighted image shows grade 1, 1 (observer 1) and 1, 1 (observer 2) at L4-5 and grade 1, 1 (observer 1) and 1, 1 (observer 2) at L5-S1 of interface between upper and lower margin of the cage and bone marrow. (b) Sagittal FSE-SEMAC T2 weighted image shows grade 3, 2 (observer 1) and 2, 2 (observer 2) at L4-5 and grade 3, 2 (observer 1) and 2, 2 (observer 2) at L5-S1 of interface between upper and lower margin of the cage and bone marrow. Diagnostic confidence level was grade 3 (inconclusive, observer 1) and 2 (suspiciously absent, observer 2) in FSE T2 weighted sagittal image, but after application of FSE-SEMAC, diagnostic confidence level was enhanced to grade 2 (suspiciously absent, observer 1) and grade 1 (definitely absent, observer 2), respectively.

The visibility of the neural foramen, nerve root, bone-inter-body cage interface, and bone-pedicle screw interface were graded based on the following criteria: grade 0, not visible; grade 1, visible area less than 1/2 of anatomical structure; grade 2, visible area more than 1/2 of anatomical structure, but slightly affected by metallic artifact; grade 3, completely visible (Figs 2–4). The central canal was divided into 4 areas; right upper(1), left upper(2), right lower(3) and left lower(4), according to anterior-posterior and right-left midlines (Fig 2A). The 4-point-grading system mentioned above was applied for each quadrant of the central canal and these measurements were averaged to grade central canal visibility. Back muscle visibility was graded on a 6-point scale on each side of the body, and the average of the two was regarded as the visibility grade. This 6-point scale was applied to evaluate back muscle visibility because the areas that were affected by metal artifacts could have been smaller than those by other anatomic structures. The grading scale was applied as follows: grade 0, no visible back muscle; grade 1, visible area less than 25% of anatomical structure; grade 2, visible area between 25%-50% of anatomical structure; grade 3, visible area between 50%-75% of anatomical structure; grade 4, visible area more than 75% of expected area, but slightly affected by metallic artifact; grade 5, completely visible.

### Semiqualitative Analysis

For the semi qualitative evaluation, both reviewers evaluated conventional FSE sequence and FSE-SEMAC sequence independently in the same way for semiquantitative analysis. The reviewers then recorded their confidence level for the diagnosis of the post-operative complications, including central canal stenosis, neural foraminal stenosis, non-union of the inter-body cage, epidural lesion, and para-spinal lesion and graded them as follows: grade 1, definitely absent; grade 2, suspiciously absent; grade 3, inconclusive; grade 4, suspiciously present; grade 5, definitely present. Central canal stenosis is defined as the obliteration of the CSF space in front of the cauda equina in the dural sac on T2-weighted axial images, regardless of the severity of stenosis [15]. We diagnosed neural foraminal stenosis in images classified as grade 2 or higher, according to the modified classification by Kunogi and Hasue [16]. In cases of T2 hyper-intensity or definite demonstration of a gap on T1 contrast-enhanced image between the bone and the inter-body cage, we presumed non-union of the inter-body cage. Epidural or para-spinal lesions included fluid collections such as hematomas or postoperative fluid collection, epidural scar formation, or postoperative infection including meningitis, arachnoiditis, superficial or deep wound infection, diskitis, abscess formation, and osteomyelitis [17].

### Statistical Analysis

For semi-quantitative evaluation of the visibility of six anatomical structures, inter-observer agreements were assessed by the means of weighted kappa values: slight, 0.20; fair, 0.21–0.40; moderate, 0.41–0.60; substantial, 0.61–0.80; and near perfect, 0.81–1.00 [18]. Generalized estimating equation (GEE) regression analysis was performed to evaluate differences in visibility scores of each anatomical structure between conventional FSE and FSE-FSE-SEMAC sequence. A *P*-value < .05 was considered statistically significant. All statistical analyses were executed using SAS version 9.3 (SAS Institute, Cary, NC).

We calculated the distribution of grades for the diagnosis of the post-operative complications to evaluate the confidence level qualitatively. For the distinction between conventional FSE and FSE-SEMAC in statistical data, we classified confidence levels into two groups. The grade 2 (suspiciously absent), grade 3 (inconclusive), and 4 (suspiciously present) in original grading system were classified as group 0 (not sure). Grade 1 (definitely absent) and grade 5 (definitely present) were classified as Group 1(sure). Generalized estimating equation (GEE) regression analysis was performed due to qualitative evaluation of the confidence level.

## Results

For the semi-quantitative evaluation, 245 neural foramina from 54 patients and 126 bone-inter-body cage interfaces from 63 cages in 40 patients were used in the sagittal plane. One neural foramen was excluded from sagittal analysis owing to recurrent and residual mass of a malignant peripheral nerve sheath tumor in the left paravertebral area of the target level. Evaluated neural foramina ranged from 1 to 4, with a mean of 2.27 ± 0.53 (one neural foramen: 4 patients, two neural foramina: 34 patients, three neural foramina: 13 patients and four neural foramina: 3 patients). 52 patients out of 54 underwent inter-body fusion, but 12 cases had inter-body fusion with allograft bone only. 40 cases were included to sagittal evaluation of bone-cage interface. We evaluated 1 case at the L2-3 level, 11 cases at the L3-4 level, 33 cases at the L4-5 level, and 18 cases at the L5-S1 level. We investigated a maximum of two spinal levels in each MR image, because the axial plane of FSE-SEMAC was obtained only in two spinal levels due to its relatively long acquisition time. In eight cases among 54 patients, one spinal level was analyzed due to incomplete coverage of the FSE-SEMAC sequence at the target level. We evaluated the axial plane of MR images at 100 levels of central canals, back muscles, and nerve roots in the epidural space of 54 patients. The great majority of evaluated spinal levels were L4 and L5, which account for 47 and 46 levels, respectively, followed by L3, L2 and L1. (19, 8 and 3 levels, respectively) We also analyzed the bone-implant interface of 200 pedicle screws in 100 spinal levels. The number of included pedicle screws per patient ranged from one to three, and all patients contained two pedicle screws per spinal level.

Table 2 depicts the visibility scores of six anatomical structures in post-operative spinal MR images. All six anatomical structures were seen more clearly on FSE-SEMAC than on FSE sequence in both axial and sagittal images. The difference between FSE-SEMAC and FSE was statistically significant (*P*-value < .0001). Kappa value ranged from 0.45(nerve root in epidural space in axial images) to 0.69 (neural foramen and bone interbody cage interface in sagittal images) on FSE-SEMAC. On the FSE sequence, kappa value was measured from 0.49 (bone-pedicle screw interface in sagittal images) to 0.75 (neural foramen in sagittal images). So, inter-observer agreements were moderate to substantial.

**Table 2:** Image quality scores for comparison of FSE sequence versus FSE-SEMAC sequence.

Evaluated structure	Visualization Score						k Value *		*P* Value
	Reader 1			Reader 2					
	FSE	FSE-SEMAC	*P* Value	FSE	FSE-SEMAC	*P* Value	FSE	FSE-SEMAC	
**Sagittal images**									
Neural foramen	1.76 ± 0.79	2.46 ± 0.60	< .0001	1.88 ± 0.77	2.45 ± 0.65	< .0001	0.75	0.69	< .0001
Bone-interbody cage interface	1.30 ± 0.59	2.27 ± 0.51	< .0001	1.33 ± 0.59	1.78 ± 0.63	< .0001	0.61	0.69	< .0001
**Axial images**									
Central canal	1.41 ± 0.64	1.81 ± 0.39	< .0001	1.04 ± 0.73	1.63 ± 0.58	< .0001	0.69	0.59	< .0001
Back muscle	3.12 ± 0.60	3.77 ± 0.47	< .0001	3.12 ± 0.69	3.75 ± 0.52	< .0001	0.53	0.64	< .0001
Bone-pedicle screw interface	0.57 ± 0.611	2.19 ± 0.48	< .0001	0.88 ± 0.48	1.86 ± 0.51	< .0001	0.49	0.46	< .0001
Nerve root in epidural space	1.15 ± 0.63	2.55 ± 0.59	< .0001	1.20 ± 0.66	2.50 ± 0.65	< .0001	0.60	0.45	< .0001

Values are mean ± SD.

FSE = fast spin-echo; FSE-SEMAC = fast spin echo- slice encoding for metal artefact correction.

*The weighted k values were reported as follows: slight, 0.20; fair, 0.21–0.40; moderate, 0.41–0.60; substantial, 0.61–0.80; and near perfect, 0.81–1.00.

Table 3 depicts simplified results for number of cases of post-operative complications as positive (grade 4 and 5), inconclusive (grade 3), or negative (grade 1 and 2). We judged there was 37 (reviewer 1) or 19 (reviewer 2) post-operative complications with FSE-SEMAC sequence. Compared to FSE sequence only, 7 or 2 positive cases of postoperative complication were suspected in FSE-SEMAC sequence. Both readers had 16 inconclusive interpretations with FSE alone, but reader 1 resolved all of them with FSE-SEMAC. Reader 2 resolved 14 of them (see Table 3). The suspected complications include infectious spondylitis with or without abscess formation, scar formation, hematoma or fluid collection in the epidural space and para-spinal space, neural foraminal stenosis due to screw loosening, and central canal stenosis on MR images (Figs 3 and 4). Only two cases remained inconclusive in FSE-SEMAC sequence. The general confidence to decide postoperative complications was enhanced. The confidence level for a diagnosis of neural foraminal stenosis was most commonly changed from grade 3 with FSE-SEMAC sequence (13 cases for reviewer 1, 11 cases for reviewer 2, Fig 2). Except only 7 patients, 47 patients had been available for clinical and/or radiologic follow up in our institution due to orthopedic problems including back pain itself and other medical problems. Among 47 patients, 13 patients had sustained back pain or leg pain. Although all suspected prosthesis-related complications were surgically confirmed, a total 8 patients underwent revision operation due to sustained back pain. We found 10 concordant complications with MR imaging. The surgically confirmed complications include the postoperative fluid collection in the epidural space, pedicle screw loosening, neural foraminal stenosis, central canal stenosis and bone-inter-body cage non-union. Table 4 depicts statistical difference of diagnostic confidence level between group 0 (not sure; included grade 2, 3 and 4) and 1(sure; included grade 1 and 5). Except central canal stenosis (Group 1: 101 (93.5%) in FSE only, 103 (95.4%) in FSE-SEMAC) *P*-value = .2408), diagnostic confidence level for other post-operative complications showed significant difference between two groups (*P*-value < .0000, Fig 3) in the statistical data (S1 File).

**Table 3:** Summary for diagnosis of post-operative complications.

Number of cases	Reviewer 1		Reviewer 2	
	FSE only	FSE-SEMAC	FSE only	FSE-SEMAC
Negative (grade 1 and 2)	224 (82.9%)	233 (86.2%)	237 (87.7%)	249 (92.2%)
Inconclusive (grade 3)	16 (6%)	0 (0%)	16 (5.9%)	2 (0.8%)
Positive (grade 4 and 5)	30 (11.1%)	37 (13.8%)	17 (6.4%)	19 (7.0%)

**Table 4:** Diagnostic confidence of postoperative complications.

Post-operative complication	FSE only		FSE-SEMAC		*P* value
	Group 0	Group 1	Group 0	Group 1	
Central canal stenosis	7 (6.5%)	101(93.5%)	5(4.6%)	103(95.4%)	.2408
Neural foraminal stenosis	68(63.0%)	40(37.0%)	21(19.4%)	87(80.6%)	< .0001*
Interbody non-union	56(51.8%)	52(48.2%)	17(15.7%)	91(84.3%)	< .0001*
Epidural lesion	25(23.2%)	83(76.8%)	12(11.1%)	96(88.9%)	< .0001*
Paraspinal lesion	12(11.1%)	96(88.9%)	4(3.7%)	104(96.3%)	< .0001*

Group 0 (not sure) includes grade 2 (suspiciously absent), grade 3 (inconclusive), and 4 (suspiciously present) in original grading system. Group 1(sure) includes grade 1 (definitely absent) and grade 5 (definitely present).

*All relevant data are within the paper and its Supporting Information files

## Discussion

MRI is the most valuable method for assessing spinal structures and evaluating post-operative complications, but metal artifact limits analysis of post-operative spinal imaging. Metal artifact results from the sudden difference in magnetic susceptibility between prostheses and periprosthetic tissues. Signal loss caused by dephasing, failure of fat suppression, and distortion are common types of metal artifacts. Spin echo techniques reduce signal dephasing by using a 180° refocusing pulse that reverses static field dephasing. Ultrashort echo-time sequence can help to reduce signal loss from dephasing [19]. Fat saturation is most commonly used method of fat suppression to use chemically selective saturation, but it leads to a mis-saturation pulse due to shifting of adjacent fat. Instead of fat saturation, (STIR,iterative decomposition of water,fat with echo asymmetry and least-squares estimation (IDEAL) help to avoid artifact [3,5,19,20]). There are several methods for reducing spatial distortion from artifacts such as view-angle tilting or multispectral imaging, MAVRIC and SEMAC techniques [5,21]. View-angle tilting corrects inhomogeneous perturbations in the local magnetic field in the vicinity of a metallic device by compensatory gradient. MAVRIC is a method to correct both in-plane and through-slice displacement artifacts [5]. SEMAC can robustly encode each excited slice against metal-induced field inhomogeneity by extending a view-angle-tilting (VAT) spin-echo sequence with additional z-phase encoding, leading to reduce metal artifact. However, SEMAC requires increased scanning time because greater slices are required to adequately cover the volume of interest, and it also has a reduced signal-to-noise ratio because the voxel size has been reduced [4,5].

Despite several weakness of the SEMAC technique, it is a convenient application method clinically, based on 3.0-T MR, and is effective in reducing metallic artifacts when evaluating post-operative spinal imaging [2,4,8,9,19]. Recent studies have focused on the visibility of anatomical structures or areas of artifact in spine, knee and hip MR imaging obtained from patients with metallic devices [2,5,7–9,20,22]. Previous reports have compared conventional FSE sequence with MAVRIC to evaluate patients who underwent hip, shoulder, or knee arthroplasty or MAVRIC-SEMAC Hybrid [23]. R. Sutter et al. published several *in vivo* articles, which found that SEMAC markedly improved detection of peri-prosthetic osteolysis over optimized standard MRI sequence in patients with total knee arthroplasty. Several articles by Lee et al. showed that SEMAC technique as it applies to spinal imaging improved the ability to visualize the neural foramina near the prosthesis, the pitches of the screw in the pedicle and the bone-prosthesis interface. These findings mean that SEMAC could be useful in visualizing bone resorption, osteolysis, peri-prosthetic fluid, infection, or prosthetic loosening in the peri-prosthetic region. However, long MR scan time is still the principal weakness of SEMAC technique in spinal MR imaging [8,10]. In our study, acquisition time of FSE-SEMAC of T2-weighted image and contrast-enhanced T1-weighted images was 310 seconds and 390 seconds, respectively. The approximate scan time of fat-saturated SEMAC sagittal T2-weighted MR protocol in the study perfomed by Lee et al. 5min 30 secs which is similar with our results, despite not exactly same MRI parameter [10].

We evaluated the added value of FSE-SEMAC sequence in the diagnosis of post-operative complications using the confidence degree of its detection as well as the visibility of adjacent anatomical structures around the metallic device in post-operative spine MR imaging with prostheses. FSE-SEMAC T2- weighted sagittal and contrast-enhanced T1- weighted axial sequence produced significantly higher scores than FSE T2-weighted and contrast-enhanced T1-weighted images for all 6 imaging findings including the neural foramen, the bone-inter-body cage interface, central canal, back muscle, bone-pedicle screw interface, and the nerve root in the epidural space (*P*-value < .0001). Although we found adequate reliability of inter-observer agreement in both reviewers, there was increased concordance on the sagittal images, with respect to axial images. We evaluated the visibility of neural foramen and bone-inter body cage in sagittal plane and central canal, back muscle, bone-pedicle screw interface and nerve root in epidural space in axial plane. We assume that the distinction of evaluated anatomical structures may be one of the reasons for the variability of lower kappa value, including axial and sagittal images difference. Although the weighted kappa value of bone-pedicle screw interface is the lowest (0.49 in FSE, 0.46 in FSE-SEMAC) among those of other anatomical structures, these fall within the moderate inter-observer agreement.

Moreover, by adding FSE-SEMAC sequence to the FSE sequence, we could decide more confidently 7 and 2 more post-operative complications. Moreover, 16 (reviewer 1) and 16 (reviewer 2) inconclusive decisions regarding five types of post-operative complications based on the FSE sequence only were turned to negative or positive complications when the FSE-SEMAC sequence was reviewed. Neural foraminal stenosis is the most commonly detected local complication, whether present or absent. These results could justify spending the time to obtain the FSE-SEMAC sequence in post-operative cases. In our study, among 47 patients who were available for follow up, 18 patients had residual or recurrent back pain after lumbar surgery which needed pain relieving procedure including epidural steroid injection or revision operation.(38.3%) The incidence of sustained back pain following lumbar surgery was reported in the range of 10% to 40% [24].

This study has, however, several limitations. First, we didn’t have surgical correlations with post-operative complications that were showed on MR images, nor did we performed clinical follow-up to determine whether management was changed. However, this study had been designed to evaluate the diagnostic confidence instead of diagnostic accuracy in which classification according to surgical findings was not necessary. Second, we only included spinal instrumentation surgery with titanium alloy pedicle screws or inter-body cages. PEEK, PE-100 or stainless steel also could have been utilized during spinal surgery. However, titanium alloy was the most commonly used material in most institutions. Third, blind analysis of conventional FSE images and FSE-SEMAC images was impractical due to the distinct differences in areas affected by metal artifact in two observers. Fourth, although inter-observer agreement showed good reliability, the measured grades of six anatomical structures based on our designed scoring method could be quite subjective. Fifth, recall bias may exist. 10 random patients for training of the semi-quantitative evaluation before using the new grading system. So we might remember and score the visibility of anatomical structure and diagnostic confidence of postsurgical complications involuntary. Sixth, this was a comparative study of conventional sequence, which was not an optimal setting to lessen metal artifact with FSE-SEMAC. Therefore, further studies require comparing FSE-SEMAC to other alternative methods to reduce metal artifact such as STIR, high readout bandwidth, view angle tilting, or other multispectral imaging should be performed.

In conclusion, FSE-SEMAC sequence significantly reduces geometric distortion by metallic implant, leading to improved assessment of anatomical structures, compared to conventional FSE sequence in 3.0-T MR. Also, higher diagnostic confidence was achieved by conventional FSE sequence added SEMAC, which provided more clear surrounding anatomical tissue around spinal prosthesis, compared to optimized traditional sequences alone.

## Supporting Information

S1 File Result file. This is raw data of this study.
(XLSX)

## Abbreviations

SEMAC: Slice-Encoding Metal Artifact Correction
FSE: Fast Spin Echo
